# An Insight into Aging, Senescence, and Their Impacts on Wound Healing

**DOI:** 10.20900/agmr20210017

**Published:** 2021-07-21

**Authors:** Rex Jeya Rajkumar Samdavid Thanapaul, Maria Shvedova, Grace Haeun Shin, Daniel S. Roh

**Affiliations:** Division of Plastic and Reconstructive Surgery, Department of Surgery, Boston University School of Medicine, Boston, MA 02118, USA

**Keywords:** aging, senescence, wound healing, senolytics, senomorphics

## Abstract

Cellular senescence has been found to have beneficial roles in development, tissue regeneration, and wound healing. However, in aging senescence increases, and the ability to properly repair and heal wounds significantly declines across multiple tissues. This age-related accumulation of senescent cells may cause loss of tissue homeostasis leading to dysregulation of normal and timely wound healing processes. The delays in wound healing of aging have widespread clinical and economic impacts, thus novel strategies to improve wound healing in aging are needed and targeting senescence may be a promising area.

## INTRODUCTION

Aging is one of the strongest risk factors for developing wound complications and chronic wounds [1]. Thus, chronic wounds such as venous leg ulcers, diabetic foot ulcers, pressure ulcers, are more frequent in older patients [2], which imposes a large economic burden on healthcare [3]. As a result, the cost of caring for and managing non-healing ulcers or poorly managed wounds is estimated to be $25 billion per year [4], and care for chronic wounds in older adults costs about $10 billion annually [5]. Considering clinical and socioeconomic burdens of wounds in the aged population, research, and development of interventions to prevent chronic wounds and improve wound healing in the geriatric population becomes a significant priority.

## AGING AND SENESCENCE

Aging is linked with multiple pathways of cellular dysfunction, including genomic instability, inflammation, stem cell dysfunction, and cellular senescence [6,7]. Senescent cells accumulate during aging [8] through a variety of mechanisms, including replicative and premature senescence. Telomere shortening is associated with replicative senescence because it results in critically short and dysfunctional telomeres, which cells detect as double-strand breaks [9]. When cells are exposed to oxidative stress and/or DNA damage as a result of physical or chemical agents, premature senescence occurs [10]. Senescent cells are characterized by cell cycle arrest, resistance to apoptosis, chromatin remodeling, morphological changes, such as flattened shape, cell enlargement, and a disrupted nuclear membrane [11,12]. These cells have a senescence-associated secretory phenotype (SASP) which influences surrounding cells and tissues [13,14].

Senescent cells play beneficial roles in the normal development, maintenance of tissue homeostasis, and have important physiological functions during aging, such as tumor suppression by halting cell cycle progression [15]. However, senescence has also been implicated as a major cause of age-related disease and aberrant tissue repair [16].

## THE ROLE OF SENESCENCE IN NORMAL WOUND HEALING

Wound healing of the skin is characterized by three phases: inflammation, proliferation, and remodeling [17]. At different life stages, the wound healing process is altered across all phases and the speed of the wound healing process slows with aging [18]. In combination with malnutrition and accumulation of comorbidities, older age can permanently hinder the healing process leading to chronic non-healing wounds.

Senescence has been shown to play both physiological and pathological roles in the wound healing process. Senescent cells are present for a short period (between 3 and 12 days) in young mice in the wound site. Although there are important differences in murine and human wound healing, senescent cells have been detected in both wound models. These senescent cells restrict fibrosis [30] and promote divergence of myofibroblasts and therefore facilitate wound contraction and closure via SASP component PDGF-AA [20]. These short-lived senescent cells may initially enhance wound healing, but if allowed to persist, chronic senescence effects shift to a detrimental pro-inflammatory and proteolytic phenotype which limits regeneration [20,31]. Several senescence markers that arise during wound healing are cyclin-dependent kinase inhibitors (CDKI) such as p16^INK4a^ and p21^CIP1^ which among other CDKI influence wound healing (Table 1).

## SENESCENCE FEATURES IN ABNORMAL WOUND HEALING

### Delayed Wound Healing of Aging

The senescence response to wounding in aged organisms has not been fully characterized. Jiang et al found elevated and sustained p21 expression in aged (24 months old) mice after wounding which when downregulated with p21 siRNA improved aged wound healing rates [27]. Thus, sustained senescence after wounding may delay wound healing, and interventions that limit excessive senescence may be options for improving wound healing in aging.

### Chronic Wounds in Aging

An increased presence of senescent cells has been hypothesized to contribute to the pathophysiology of chronic wounds which occur more frequently in older adults. Fibroblasts, harvested from human venous ulcers and pressure ulcers, grown in cell culture have features of senescence [32,33]. Chronic wounds can also harbor pathogenic microorganisms, which can contribute to senescence by increasing ROS production in keratinocytes and promoting inflammation [34]. The chronic ulcer milieu, which is high in pro-inflammatory factors, contributes to persistent wound senescence by exacerbating inflammation and, in addition, senescent chronic wound cells can promote senescence in neighboring cells through SASP [35]. A novel mechanistic link between aging and wound healing in diabetic wounds was recently identified in which intrinsically senescent macrophages were found to promote impaired wound healing in a diabetic (db/db) mice model [36,37].

## THERAPEUTIC APPROACHES FOR SENESCENCE MANIPULATION TO IMPROVE WOUND HEALING IN AGING

Evidence for the variations between transient and chronic senescence is emerging, which has important implications for wound healing in aging. The evolving concept of transient vs. chronic senescence may provide significant new insight into the processes that occur during acute and pathological repair. Currently, transient senescence has been found to support tissue repair and wound healing whereas prolonged senescence has the opposite effect. Substances that specifically alter senescent cells offer a promising clinical tool for modulating delayed wound healing in aging and chronic wounds.

**Figure 1.**
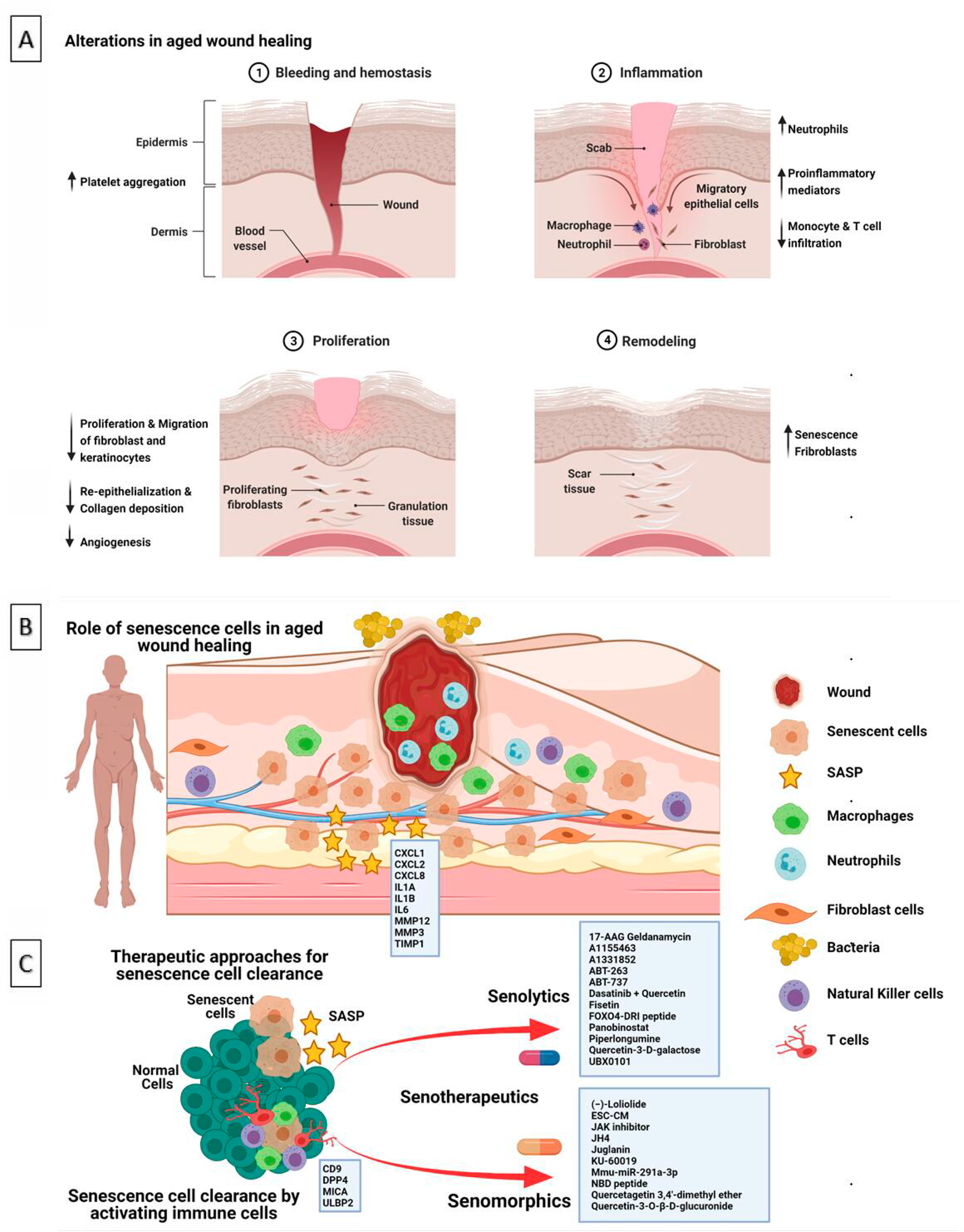
Role of aging, senescence in wound healing. **A** & **B**: Alterations and role of senescence in aged wound healing; **C**: Therapeutic approaches for senescence cell clearance. (Created in BioRender.com).

Senolytic substances selectively eliminate senescent cells and have the potential for the treatment of delayed wound healing and chronic wounds [38] (Figure 1). Xu et al. found that aged (20 months) mice improved their physical performance after receiving bi-weekly oral treatments of Dasatinib (D) and Quercetin (Q) for four months [39] and a single 3-day oral treatment of D+Q (Phase I clinical trial) was able to reduce senescence in diabetic patients' adipose tissue [40]. These findings imply that senolytic treatments can not only have immediate effects in target peripheral tissues but also overcome established tissue senescence. Senolytics could be considered in the treatment of human chronic wounds with elevated senescence levels. Senescence-oriented therapies may have the potential for the treatment of chronic wounds in the future.

Senomorphics are an alternative approach to senescence modification that inhibits SASP or SASP components without killing senescent cells [41,42]. Senomorphics are agents that can convert senescent cell phenotypes to those of non-senescent cells by disrupting senescence-related signaling pathways and inflammation [43]. There are several classes of senomorphics that suppress senescence markers or their secretory phenotype without inducing apoptosis [44]. SASP inhibitors, such as metformin and rapamycin, have been found to accelerate diabetic mice wound healing and to increase the lifespan of mice, human skin fibroblasts, and keratinocytes [45–48]. Metformin and rapamycin demonstrated essential angiogenic and rejuvenative activities via the AMPK pathway in both young and elderly skin, and local administration of metformin has promising regenerative potential in healing cutaneous wound defects [49]. The disadvantage of senomorphics is that they must be administered continuously, necessitating improved safety profiles. Chronic treatment to suppress senescence or SASP may not be desirable if there are also beneficial features of senescence [41]. Also, one must consider that the components of SASP differ between senescent cell types and making targeting SASP factors more challenging. Understanding the biological significance of various senescent cell populations and their SASP components may improve senomorphic target specificity [50]. It should be noted that proving a drugʼs senolytic or senomorphic properties is challenging and should be approached with caution.

## NATURAL COMPOUNDS THAT CAN MODIFY SENESCENCE AND WOUND HEALING PROCESS

The anti-SASP and/or senolytic activity of natural compounds are now being studied. Table 2 summarizes the effects of senolytic agents on senescent cells and wound healing. Despite the amount of in vivo and in vitro evidence on natural anti-senescence compounds, information on their safety and efficacy remains limited. Hormesis, as a health-promoting activity, can modulate the impact of natural or synthetic aging modulators as there is generally a favorable biological response to repeated low exposures of these compounds. However, the relatively high dosages necessary to induce biological effects, as well as their variable bioavailability, are two outstanding issues. Furthermore, no long-term human studies have shown the pharmacodynamics, pharmacokinetics, and hazardous consequences of an excess of natural antisenescence agents.

## CONCLUSIONS

Delays in wound healing of aging have widespread clinical and economic impacts and novel strategies to improve wound healing in aging are needed. The evidence for the role of senescence in wound healing is growing, however, how senescence impacts wound healing of aging remains to be determined. Elevated baseline senescence and defective senescent clearance mechanisms that occur in aging may be initial targets to improve healing. Developing methods to promote transient senescence response during wound healing may prove to be a beneficial therapeutic approach to accelerate wound healing in aging. Senescent cell removal and SASP reduction are potential therapeutic strategies for improving wound healing during aging.

## Figures and Tables

**Table 1.** Protein cyclin-dependent kinase inhibitors and their effect on wound healing.

CDKIs	Effects on wound healing
	reduces scarring [19]
**p15**	

	emerges early in response to cutaneous wound in young mice [20] promotes cutaneous wound healing by inducing CYR61 [21] activate responses in normal wound healing through Keratinocyte migration and re-epithelialization [22]
**p16**	high expression in chronic wounds [23,24]

	delays the onset of the proliferation phase of wound healing [25] prolongs inflammation and scarring [26] delayed wound healing in aged mice [27] enhances wound healing and skin regeneration in elderly adults [27] promotes wound repair without jeopardizing tumor suppressive function [27]
**p21**	high expression in chronic wounds [28]

	limits cell proliferation [29]
**p27**	maintains cell tissue homeostasis during adulthood [29]

**Table 2.** Effects of senolytic agents on senescent cells and wound healing.

Senolytic agents	Role on senescence and wound healing	References
**Quercetin**	Promotes senescent cell clearance in healthy tissue. Combination of quercetin and dasatinib had significant effects on health span Promotes wound healing by enhancing fibroblast proliferation and decreasing fibrosis and scarring Promotes diabetic wound healing by altering macrophage polarization Demethylates the p16ink4a gene promoter	[39,51–53]
**Epigallocatechin Gallate**	Decreased the level of acetylated p53 Promotes wound healing through targeting notch signaling	[54,55]
**Resveratrol**	Protect human lung fibroblasts from senescence caused by a high glucose environment Enhances healing by improving cell proliferation and migration	[56,57]
**ABT263**	Clears senescent cells induced by DNA damage in the lung Removes senescent cells induced by p53 activation in the epidermis via transgenic p14(ARF) Involvement of apoptosis-related signal pathway in the senescent cell elimination process	[58,59]
**KU-60019**	Promotes cutaneous wound healing in aged mice by inhibiting ATM kinase	[60]
**Mmu-miR-291a-3p**	Promotes excisional skin wound healing in aged mice by targeting the TGFBR2/p21 pathway	[61]
